# Valor do Diâmetro do Átrio Esquerdo com Escore CHA2DS2-Vasc na Predição da Trombose Atrial Esquerda/Trombose de Apêndice Atrial Esquerdo na Fibrilação Atrial Não Valvar

**DOI:** 10.36660/abc.20190492

**Published:** 2021-01-13

**Authors:** Yu Zhang, Yi-Qiang Yuan

**Affiliations:** 1 Cardiovascular Hospital of Zhengzhou Cardiovascular Hospital of Zhengzhou Zhengzhou China Cardiovascular Hospital of Zhengzhou, Zhengzhou - China

**Keywords:** Fibrilação Atrial Não Valvar, Acidente Vascular Cerebral, Avaliação de Risco, Escore de Propensão, Átrio Esquerdo, Apêndice Atrial, Trombose

## Abstract

**Fundamentos:**

A fibrilação atrial é a arritmia persistente mais comum e é o principal fator que leva ao tromboembolismo.

**Objetivo:**

Investigar o valor do diâmetro do átrio esquerdo combinado com o escore CHA2DS2-VASc na predição da trombose atrial esquerda/trombose de apêndice atrial esquerdo na fibrilação atrial não valvar.

**Métodos:**

Trata-se de estudo retrospectivo. 238 pacientes com fibrilação atrial não valvar foram selecionados e divididos em dois grupos: trombose e não trombose. Determinou-se o escore CHA2DS2-VASc. Valores de p<0,05 foram considerados estatisticamente significativos.

**Resultados:**

A análise de regressão logística multivariada revelou que histórico de acidente vascular cerebral/ataque isquêmico transitório, doença vascular, escore CHA2DS2-VASc, DAE, DDFVE e FEVE foram fatores de risco independentes para trombose atrial esquerda/trombose de apêndice atrial esquerdo (p<0,05). A análise da curva ROC ( *Receiver Operating Characteristic* ) revelou que a área sob a curva para o escore CHA2DS2-VASc na predição de trombose atrial esquerda/trombose de apêndice atrial esquerdo foi de 0,593 quando o escore CHA2DS2-VASc foi ≥3 pontos, e a sensibilidade e especificidade foram 86,5% e 32,6%, respectivamente, enquanto a área sob a curva para o DAE na predição de trombose atrial esquerda/trombose de apêndice atrial esquerdo foi 0,786 quando o DAE foi ≥44,17 mm, e a sensibilidade e especificidade foram 89,6% e 60,9%, respectivamente. Entre os diferentes grupos CHA2DS2-VASc, a taxa de incidência de trombose atrial esquerda/trombose de apêndice atrial esquerdo em pacientes com DAE ≥44,17 mm foi maior do que em pacientes com DAE <44,17 mm (p <0,05).

**Conclusão:**

O escore CHA2DS2-VASc e o DAE estão correlacionados com a trombose atrial esquerda/trombose de apêndice atrial esquerdo na fibrilação atrial não valvar. Para pacientes com escore CHA2DS2-VASc de 0 ou 1, quando o DAE é ≥44,17 mm, o risco de trombose atrial esquerda/trombose de apêndice atrial esquerdo permaneceu alto. (Arq Bras Cardiol. 2020; [online].ahead print, PP.0-0)

## Introdução

A fibrilação atrial (FA) é a arritmia persistente mais comum e é o principal fator que leva ao tromboembolismo.^1^ Nos últimos anos, com o envelhecimento da população na China, a incidência dessa doença aumentou.^2^ Portanto, essa doença representa uma séria ameaça à vida e à saúde das pessoas. Quando a FA ocorre, o átrio cardíaco não pode se contrair regularmente e efetivamente e o fluxo sanguíneo diminui, o que aumenta muito o risco de trombose atrial esquerda/trombose de apêndice atrial esquerdo,^3^ e a trombose atrial esquerda/trombose de apêndice atrial esquerdo aumenta ainda mais o risco de episódios de tromboembolismo.^4^ Portanto, a avaliação científica da trombose atrial esquerda/trombose de apêndice atrial esquerdo é de grande importância para orientar o tratamento e melhorar o prognóstico dos pacientes. O CHA2DS2-VASc é um escore atualmente e amplamente usado para avaliar o risco de acidente vascular cerebral em pacientes com FA não valvar, e desempenha um papel importante na determinação de fatores de alto risco e orientação do tratamento.^5^ No entanto, o escore depende principalmente do registro do histórico do paciente. Um estudo revelou que ^6^ o tamanho do átrio esquerdo estava intimamente relacionado à trombose atrial esquerda/trombose de apêndice atrial esquerdo. No entanto, não está claro se o diâmetro do átrio esquerdo combinado com o escore CHA2DS2-VASc pode melhorar os resultados preditivos de trombose atrial esquerda/trombose de apêndice atrial esquerdo. O objetivo deste estudo foi analisar os fatores relacionados à trombose atrial esquerda/trombose de apêndice atrial esquerdo em pacientes com FA não valvar e explorar o valor do diâmetro do átrio esquerdo combinado com o escore CHA2DS2-VASc na predição de trombose atrial esquerda/trombose de apêndice atrial esquerdo, a fim de fornecer referências para a prática clínica.

## Dados e métodos

### Dados gerais

Trata-se de um estudo retrospectivo. Os dados foram coletados em prontuários médicos. No total, 238 pacientes com FA não valvar, hospitalizados no Zhengzhou Cardiovascular Hospital de fevereiro de 2012 a março de 2017, foram incluídos no estudo. Critérios de inclusão: (1) pacientes diagnosticados por eletrocardiograma (ECG) ou ECG dinâmico; (2) pacientes submetidos à ecocardiografia transesofágica. Critérios de exclusão: (1) pacientes com cardiopatia reumática, FA valvar e FA paroxística; (2) pacientes com infarto agudo do miocárdio e insuficiência cardíaca aguda descompensada em 90 dias e pacientes com histórico de cirurgia cardíaca; (3) pacientes com embolia pulmonar, trombose venosa profunda, histórico de administração de anticoagulantes, como varfarina e rivaroxabana, ou hipolipemiantes, como estatinas; (4) pacientes com tumores malignos, hipertireoidismo e disfunção hepática e renal grave. Esse estudo foi aprovado pelo Comitê de Ética do nosso hospital. Todos os pacientes forneceram consentimento informado assinado.

## Método

### Aquisição de dados clínicos

Foram coletadas as seguintes informações de todos os pacientes: sexo, idade, curso da FA, tabagismo e alcoolismo, histórico de doença crônica, altura e peso. Calculou-se o índice de massa corporal (IMC). Além disso, glicemia em jejum (GJ), colesterol total (CT), triglicerídeos (TG), colesterol de lipoproteína de baixa densidade (LDL-c), colesterol de lipoproteína de alta densidade (HDL-c), contagem de plaquetas (Plt), ácido úrico sérico (AU) e outros indicadores bioquímicos foram coletados.

### Ecocardiografia transtorácica e ecocardiografia transesofágica

Todos os exames foram realizados por um ultrassonografista experiente com o título de Médico Chefe em nosso hospital. Todos os pacientes assinaram o termo de consentimento informado antes do exame. Para realizar o exame, utilizou-se um aparelho diagnóstico de ultrassom Doppler colorido Philips iE33. Realizou-se ecocardiografia transtorácica de rotina, e a frequência da sonda foi de 2,5 MHz. Foram medidos o diâmetro atrial esquerdo (DAE), a dimensão diastólica final do ventrículo esquerdo (DDFVE) e a fração de ejeção do ventrículo esquerdo (FEVE). Posteriormente, administrou-se anestesia faríngea local com lidocaína. A seguir, a sonda foi colocada no esôfago até a localização do coração, e a frequência da sonda foi de 5,0 MHz. As seções do átrio esquerdo e do apêndice atrial esquerdo foram continuamente observadas para determinar se o trombo estava presente no átrio esquerdo/apêndice atrial esquerdo. Esses pacientes foram divididos em dois grupos: grupo trombose e grupo não trombose.

### Escore CHA2DS2-VASc

O escore CHA2DS2-VASc foi calculado de acordo com os dados clínicos básicos dos pacientes:^7^ (1) principais fatores de risco (2 pontos por item): idade ≥75 anos, AVC isquêmico e ataque isquêmico transitório; (2) fatores secundários (1 ponto por item): mulheres, com idades entre 65 e 74 anos, hipertensão, diabetes, doença vascular e insuficiência cardíaca crônica; (3) escore mais baixo de 0 e escore mais alto de 9. Quanto maior o escore, maior a possibilidade de trombose.

### Análise estatística

Para o agrupamento de dados e análises estatísticas, utilizou-se o programa estatístico SPSS versão 21.0. Os dados contínuos foram expressos como média±desvio padrão (x±DP) e comparados entre dois grupos usando o teste *t* de Student não pareado de distribuição normal, e o teste de Kolmogorov-Smirnov (K-S) foi usado para distribuição normal. Os dados categóricos foram expressos em taxas (%) e comparados entre dois grupos usando o teste *X2* . Realizou-se a análise de regressão logística multivariada para analisar os fatores relacionados que afetaram a trombose atrial esquerda/trombose de apêndice atrial esquerdo. Utilizou-se a curva ROC para analisar os resultados preditivos do diâmetro atrial esquerdo e do escore CHA2DS2-VASc para trombose atrial esquerda/trombose de apêndice atrial esquerdo. Valores de p<0,05 foram considerados estatisticamente significativos.

## Resultados

### Trombose atrial esquerda/trombose de apêndice atrial esquerdo

No total, 238 pacientes com FA não valvar foram incluídos neste estudo. Entre esses pacientes, 151 pacientes eram do sexo masculino e 87 do sexo feminino, e a idade desses pacientes variou de 29 a 86, com média de 61,1±12,4 anos. Nesses 238 pacientes, ocorreu trombose atrial esquerda/trombose de apêndice atrial esquerdo em 46 pacientes, e a incidência foi de 19,3%.

### Comparação de dados clínicos entre o grupo trombose e o grupo não trombose

Diferenças de sexo, IMC, curso de FA, proporções de pacientes com histórico de tabagismo e consumo de álcool, proporções de pacientes com diabetes e doença cardíaca coronariana, escore CHA2DS2-VASc, GJ, CT, TG, LDL-c, Plt, AU e terapia medicamentosa entre o grupo trombose e o grupo não trombose não foram estatisticamente significativas (p>0,05). No grupo trombose, a proporção de pacientes com idade ≥75, a proporção de pacientes com hipertensão, a proporção de pacientes com insuficiência cardíaca, a proporção de pacientes com histórico de acidente vascular cerebral/ataque isquêmico transitório, a proporção de pacientes com histórico de doença vascular e o escore CHA2DS2-VASc, DAE e DDFVE foram maiores do que aqueles no grupo não trombose, enquanto o HDL-c e FEVE foram menores do que aqueles no grupo não trombose, e todas as diferenças foram estatisticamente significativas (p<0,05, Tabela 1 ).

**Tabela 1:** – Dados clínicos do grupo trombose e do grupo não trombose

Índice	Grupo trombose (n=46)	Grupo não trombose (n=192)	*t* /χ^**2**^	p
**Idade (n, %) **				
<65 anos	18 (39,1)	112 (58,3)		
65–74 anos	15 (32,6)	62 (32,3)		
≥75 anos	13 (28,3)	18 (9,4)	12,668	0,002
Sexo (M/F)	32 (69,6)	119 (62,0)	0,921	0,337
IMC (kg/m^2^)	26.82±3,70	25.94±3,01	1,696	0,091
Anos de AF (A)	4.69±1,69	5.10±1,38	1,718	0,087
Tabagismo (n, %)	18 (39,1)	71 (37,0)	0,921	0,337
Alcoolismo (n, %)	11 (23,9)	36 (18,8)	0,624	0,429
Hipertensão arterial (n, %)	32 (69,6)	91 (47,4)	7,304	0,007
Diabetes mellitus (n, %)	8 (17,4)	44 (22,9)	0,664	0,415
Doença coronariana (n, %)	4 (8,7)	8 (4,2)	1,590	0,207
Insuficiência cardíaca, (n, %)	7 (15,2)	6 (3,1)	10,508	0,001
Acidente vascular cerebral/Ataque isquêmico transitório (n, %)	17 (37,0)	11 (5,7)	101,138	0,000
Doença vascular (n, %)	22 (47,8)	51 (26,6)	7,890	0,005
GJ (mmol/L)	5,72±0,86	6,13±1,43	1,832	0,068
CT (mmol/L)	4,82±0,96	4,66±0,98	1,036	0,301
TG (mmol/L)	1,84±1,02	1,68±0,92	1,055	0,292
LDL-c (mmol/L)	3,00±0,54	2,96±0,86	0,298	0,766
HDL-c (mmol/L)	0,99±0,18	1,16±0,31	3,458	0,001
Plt (×10^9^/L)	209,08±34,45	214,43±41,26	0,815	0,416
AU (μmol/L)	333,70±64,68	342,74±70,08	0,798	0,426
Escore CHA2DS2-VASc	2,26±1,90	1,64±1,48	2,428	0,016
Grupo CHA2DS2-VASc (n, %)			2,635	0,268
Escore 0	8 (17,4)	55 (28,6)		
Escore 1	14 (30,4)	56 (29,2)		
Escores ≥2	24 (52,2)	81 (42,2)		
DAE (mm)	45,81±6,16	38,55±5,00	6,118	0,000
DDFVE (mm)	51,35±4,38	48,53±4,11	4,133	0,000
FEVE (%)	57,05±10,50	61,84±9,17	3,092	0,002
**Tratamento medicamentoso (n, %) **				
Betabloqueadores	15 (32,6)	54 (28,1)	0,362	0,547
IECA/BRA	21 (45,7)	75 (39,1)	0,670	0,413

*MC: índice de massa corporal; GJ: glicemia em jejum; CT: colesterol total; TG: triglicerídeos; LDL-c: colesterol de lipoproteína de baixa densidade; HDL-C: colesterol de lipoproteína de alta densidade; Plt: contagem de plaquetas; AU: ácido úrico sérico; DAE: diâmetro atrial esquerdo; DDFVE: dimensão diastólica final do ventrículo esquerdo; FEVE: fração de ejeção do ventrículo esquerdo; IECA: inibidor da enzima de conversão da angiotensina; BRA: bloqueadores dos receptores de angiotensina.*

### Fatores relacionados que afetam a trombose atrial esquerda/trombose de apêndice atrial esquerdo

Com a determinação de se a trombose atrial esquerda/trombose de apêndice atrial esquerdo existia como uma variável dependente, e as variáveis com um valor de p<0,10 como variáveis independentes, realizou-se análise de regressão logística multivariada. Os resultados revelaram que o histórico de acidente vascular cerebral/ataque isquêmico transitório, doença vascular, escore CHA2DS2-VASc, DAE, DDFVE e FEVE foram fatores de risco independentes para trombose atrial esquerda/trombose de apêndice atrial esquerdo (p<0,05, Tabela 2 ).

**Tabela 2:** – Fatores relacionados a trombo em átrio esquerdo ou apêndice atrial esquerdo

Índice	B	EP	Wals χ^**2**^	p	OR (CI: 95%)
AVC/Ataque isquêmico transitório	3,597	1,165	9,528	0,002	36,498 (3,718∼358,322)
Doença vascular	1,280	0,574	4,979	0,026	3,597 (1,168∼11,071)
HDL-c	2,574	1,021	6,354	0,012	13,124 (1,773∼97,142)
Escore CHA2DS2-VASc	-0,441	0,171	6,610	0,010	0,644 (0,460∼0,901)
DAE	-0,246	0,058	18,025	0,000	0,782 (0,698∼0,876)
DDFVE	-0,173	0,063	7,432	0,006	0,841 (0,743∼0953)
FEVE	0,066	0,027	5,925	0,015	1,068 (1,013∼1,126)

*AVC: acidente vascular cerebral; HDL-C: colesterol de lipoproteína de alta densidade; DAE: diâmetro atrial esquerdo; DDFVE: dimensão diastólica final do ventrículo esquerdo; FEVE: fração de ejeção do ventrículo esquerdo*

### O valor do DAE e do escore CHA2DS2-VASc na predição de trombose atrial esquerda/trombose de apêndice atrial esquerdo

**Figura 1:**
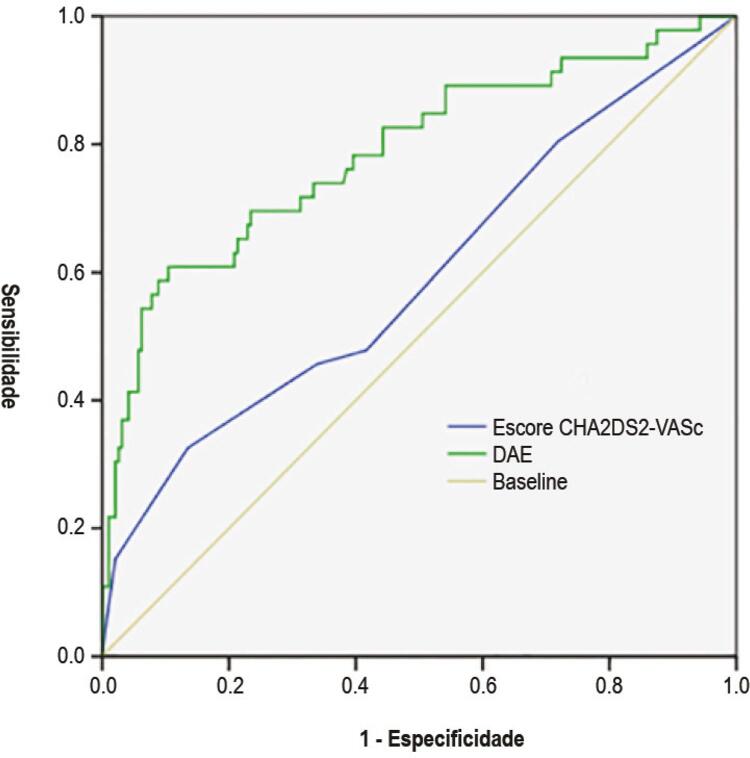
– A análise da área sob a curva ROC revelou que quando o escore CHA2DS2-VASc foi usado para predizer a trombose atrial esquerda/trombose de apêndice atrial esquerdo, a área sob a curva foi de 0,593 (IC 95%: 0,495–0,690). Quando o escore CHA2DS2-VASc foi ≥3, a sensibilidade e a especificidade foram de 86,5% e 32,6%, respectivamente. Quando o DAE foi usado para predizer a trombose atrial esquerda/trombose de apêndice atrial esquerdo, a área sob a curva foi de 0,786 (IC 95%: 0,704–0,868). Quando o DAE foi >44,17 mm, a sensibilidade e a especificidade foram 89,6% e 60,9%, respectivamente.

A análise da área sob a curva ROC revelou que quando o escore CHA2DS2-VASc foi usado para predizer a trombose atrial esquerda/trombose de apêndice atrial esquerdo, a área sob a curva foi de 0,593 (IC 95%: 0,495–0.690). Quando o escore CHA2DS2-VASc foi ≥3, a sensibilidade e a especificidade foram de 86,5% e 32,6%, respectivamente. Quando o DAE foi usado para prever trombose atrial esquerda/trombose de apêndice atrial esquerdo, a área sob a curva foi de 0,786 (IC de 95%: 0,704–0,868). Quando o DAE foi ≥44,17 mm, a sensibilidade e a especificidade foram 89,6% e 60,9%, respectivamente ( Figura 1 ).

### Efeito do DAE no risco de trombose atrial esquerda/trombose de apêndice atrial esquerdo em pacientes nos diferentes grupos CHA2DS2-VASc

Nos diferentes grupos CHA2DS2-VASc, a incidência de trombose atrial esquerda/trombose de apêndice atrial esquerdo em pacientes com DAE ≥44,17 mm foi maior do que em pacientes com DAE <44,17 mm, e a diferença foi estatisticamente significativa (p<0,05, Tabela 3 ).

**Tabela 3:** – Efeito do diâmetro do átrio esquerdo sobre o risco de trombose atrial esquerda/trombose de apêndice atrial esquerdo em pacientes com diferentes grupos de CHA2DS2-VASc (mm)

Grupo CHA2DS2-VASc	n	Trombo de átrio esquerdo/trombo de apêndice atrial esquerdo (n,%)		OR	χ^**2**^	p
		DAE≥44,17	DAE<44,17			
Escore 0	63	6/12 (50,0)	3/51 (5,9)	8,500 (IC 95%: 1,856∼38.938)	9,524	0,002
Escore 1	73	11/16 (68,8)	4/57 (7,0)	9,797 (IC 95%: 2,747∼34.943)	15,466	0,000
Escore ≥2	102	11/19 (57,9)	11/83 (13,3)	4,368 (IC 95%: 1,651∼11.559)	9,712	0,002

## Discussão

Como o tipo de arritmia mais comum em Departamentos de Clínica Médica Cardiovascular, a FA é um fator de risco que leva ao tromboembolismo^8^. Em comparação com a população não FA, o risco de AVC em pacientes com FA aumenta cinco vezes.^9^ Além disso, um estudo revelou que^10^ o trombo que causou AVC em pacientes com FA veio principalmente do átrio esquerdo/apêndice atrial esquerdo. A trombose atrial esquerda/trombose de apêndice atrial esquerdo é um fator de risco independente para acidente vascular cerebral em pacientes com FA não valvar.^11^ Isso pode aumentar significativamente o risco de eventos tromboembólicos e é um indicador direto de terapia anticoagulante em pacientes com FA.^12^ Portanto, a detecção precoce da trombose atrial esquerda/trombose de apêndice atrial esquerdo ou fatores de alto risco para trombose atrial esquerda/trombose de apêndice atrial esquerdo é de grande importância para orientar o tratamento e melhorar o prognóstico de pacientes com FA. Neste estudo, 238 pacientes com FA, que não receberam anticoagulação e terapia hipolipemiante, foram incluídos. Os resultados da ecocardiografia transesofágica revelaram que a incidência de trombose atrial esquerda/trombose de apêndice atrial esquerdo foi de 19,3%. Essa porcentagem se assemelha aos 18,6% relatados por Shuanglun Xie et al.,^13^ e aos 20,7% relatados por Weiwei Fu et al.^14^ Esses resultados revelam que a incidência de trombose atrial esquerda/trombose de apêndice atrial esquerdo é relativamente alta em pacientes com FA sem anticoagulação e terapia hipolipemiante.

O escore CHA2DS2-VASc foi estabelecido otimizando ainda mais o escore CHADS2, que é um método clínico comumente usado para avaliar o risco de acidente vascular cerebral em pacientes com FA atualmente, sendo também usado para orientar o tratamento clínico.^15^ Um estudo revelou que^16^ um escore CHA2DS2-VASc ≥2 é um fator de risco independente para trombose atrial esquerda/trombose de apêndice atrial esquerdo em pacientes com FA. O presente estudo revelou que o escore CHA2DS2-VASc foi maior no grupo trombose do que no grupo não trombose. No entanto, a diferença na distribuição dos escores CHA2DS2-VASc entre os dois grupos não foi estatisticamente significativa. A análise univariada e multivariada revelou que o escore CHA2DS2-VASc é um fator de risco independente para trombose atrial esquerda/trombose de apêndice atrial esquerdo. Além disso, esses resultados revelam que o escore CHA2DS2-VASc está correlacionado com a trombose atrial esquerda/trombose de apêndice atrial esquerdo. A análise da área sob a curva ROC revelou uma área sob a curva de 0,593 (IC 95%: 0,495–0.690). Quando o escore CHA2DS2-VASc foi ≥3, a sensibilidade e a especificidade foram de 86,5% e 32,6%, respectivamente. Esses resultados mostraram que, para pacientes com escore CHA2DS2-VASc ≥3, a possibilidade de trombose atrial esquerda/trombose de apêndice atrial esquerdo deve ser altamente alertada. No entanto, este estudo também revelou que quando o escore CHA2DS2-VASc era 0 ou 1, a trombose atrial esquerda/trombose de apêndice atrial esquerdo ainda ocorria em 9 e 15 pacientes, respectivamente. Além disso, esses resultados revelaram que, para pacientes de baixo risco com escore CHA2DS2-VASc de 0 ou 1, ainda havia risco de acidente vascular cerebral. Esses resultados sugeriram que o escore CHA2DS2-VASc tem algumas limitações na predição de trombose atrial esquerda/trombose de apêndice atrial esquerdo.

Um estudo revelou que^17^ alterações morfológicas no átrio esquerdo e no apêndice atrial esquerdo podem aumentar o risco de tromboembolismo em pacientes com FA. Quando a FA ocorre, quanto maior o átrio cardíaco, mais facilmente se forma a trombose.^18^ Neste estudo, comparou-se o DAE em pacientes com FA. Os resultados revelaram um DAE maior no grupo trombose do que no grupo não trombose, sendo um fator de risco independente para trombose atrial esquerda/trombose de apêndice atrial esquerdo. A análise da curva ROC revelou que quando o DAE foi usado para predizer a trombose atrial esquerda/trombose de apêndice atrial esquerdo, a área sob a curva foi 0,786 (IC de 95%: 0,704–0,868), e quando o DAE era ≥44,17 mm, a sensibilidade e a especificidade eram 89,6% e 60,9%, respectivamente. Esses resultados revelaram que o tamanho do DAE se correlacionou com a trombose atrial esquerda/trombose de apêndice atrial esquerdo. Portanto, quando o DAE era ≥44,17 mm, apresentava boa sensibilidade e especificidade na predição de trombose atrial esquerda/trombose de apêndice atrial esquerdo. Neste estudo, usamos o DAE como o índice para prever trombose atrial esquerda/trombose de apêndice atrial esquerdo. Recentemente, o volume atrial esquerdo tem sido usado como medida do aumento atrial esquerdo.^19^ Esse índice pode ser incluído em estudos futuros. Neste estudo, os pacientes foram estratificados de acordo com o escore CHA2DS2-VASc, a fim de analisar o efeito do DAE na trombose atrial esquerda/trombose de apêndice atrial esquerdo. Esses resultados revelaram que, independentemente de o escore CHA2DS2-VASc ser 0, 1 ou ≥2, um DAE ≥44,17 mm aumentou significativamente o risco de trombose atrial esquerda/trombose de apêndice atrial esquerdo. Esses resultados revelaram que uma avaliação adicional do DAE com base no escore CHA2DS2-VASc seria útil para avaliar o risco de trombose atrial esquerda/trombose de apêndice atrial esquerdo e orientar a terapia de anticoagulação.

No entanto, considerando que este é um estudo unicêntrico e com amostra pequena, pode haver algumas deficiências na representatividade da amostra. Portanto, estudos de coorte multicêntricos e com amostras maiores são necessários para esclarecer ainda mais a relação entre o escore CHA2DS2-VASc e o DAE na predição de trombose atrial esquerda/trombose de apêndice atrial esquerdo e orientação da terapia de anticoagulação.

## Conclusão

Em resumo, o escore CHA2DS2-VASc e o DAE estão correlacionados à trombose atrial esquerda/trombose de apêndice atrial esquerdo em pacientes com FA não valvar. Para pacientes com escore CHA2DS2-VASc de 0 ou 1, o tamanho do DAE deve ser considerado. Quando o DAE era ≥44,17 mm, o risco de trombose atrial esquerda/trombose de apêndice atrial esquerdo ainda é relativamente alto, sendo necessário conduzir terapias de anticoagulação adicionais.
