# Supplementary material for: Valor do Diâmetro do Átrio Esquerdo com Escore CHA2DS2-Vasc na Predição da Trombose Atrial Esquerda/Trombose de Apêndice Atrial Esquerdo na Fibrilação Atrial Não Valvar
Source: Arq Bras Cardiol. 2021 Jan 13;116(2):325–31. [Article in Portuguese] doi: 10.36660/abc.20190492 (PMC7909979; doi:10.36660/abc.20190492)
Supplement: Translation

# Translation: Value of left atrial diameter with CHA2DS2-VASc score in predicting left atrial/left atrial appendage thrombosis in non-valvular atrial fibrillation

**Authors:** Yu Zhang, Yi-Qiang Yuan

**Affiliations:** 1 China Cardiovascular Hospital of Zhengzhou, Zhengzhou - China

**Keywords:** Atrial Fibrillation Non Valvar, Stroke, Risk Assessment, Propensity Score, Heart Atria, Atrial Appendage

## Abstract

**Background:**

Atrial fibrillation is the most common persistent arrhythmia, and is the main factor that leads to thromboembolism.

**Objective:**

To investigate the value of left atrial diameter combined with CHA2DS2-VASc score in predicting left atrial/left atrial appendage thrombosis in non-valvular atrial fibrillation.

**Methods:**

This is a retrospective study. 238 patients with non-valvular atrial fibrillation were selected and divided into two groups: thrombosis and non-thrombosis. CHA2DS2-VASc score was determined. P<0.05 was considered statistically significant.

**Results:**

Multivariate logistic regression analysis revealed that the history of stroke/transient ischemic attack, vascular disease, CHA2DS2-VASc score, left atrial diameter (LAD), left ventricular end-diastolic dimension (LVEDD) and left ventricular ejection fraction (LVEF) were independent risk factors for left atrial/left atrial appendage thrombosis (p<0.05). Receiver operating characteristic curve analysis revealed that the area under the curve for the CHA2DS2-VASc score in predicting left atrial/left atrial appendage thrombosis was 0.593 when the CHA2DS2-VASc score was ≥3 points, and sensitivity and specificity were 86.5% and 32.6%, respectively, while the area under the curve for LAD in predicting left atrial/left atrial appendage thrombosis was 0.786 when LAD was ≥44.17 mm, and sensitivity and specificity were 89.6% and 60.9%, respectively. Among the different CHA2DS2-VASc groups, the incidence rate of left atrial/left atrial appendage thrombosis in patients with LAD ≥44.17 mm was higher than patients with LAD <44.17 mm (p<0.05).

**Conclusion:**

CHA2DS2-VASc score and LAD are correlated with left atrial/left atrial appendage thrombosis in non-valvular atrial fibrillation. For patients with a CHA2DS2-VASc score of 0 or 1, when LAD is ≥44.17 mm, the risk for left atrial/left atrial appendage thrombosis remained high. (Arq Bras Cardiol. 2020; [online].ahead print, PP.0-0)

## Introduction

Atrial fibrillation (AF) is the most common persistent arrhythmia, and is the major factor that leads to thromboembolism.^1^In recent years, with the aging of the population in China, the incidence of this disease has increased.^2^Therefore, this disease represents a serious threat to people’s life and health. When AF occurs, the cardiac atrium cannot regularly and effectively constrict, and blood flow slows down, which greatly increases the risk of left atrial/left atrial appendage thrombosis,^3^and left atrial/left atrial appendage thrombosis further increases the risk of thromboembolism events.^4^Therefore, scientific evaluation of left atrial/left atrial appendage thrombosis is of great significance for guiding treatment and improving the prognosis of patients. CHA2DS2-VASc is a presently and widely used scoring system to assess the risk of stroke in patients with non-valvular AF, and plays an important role in determining high-risk factors and guiding treatment.^5^However, the scoring system relies mainly on patient’s history record. A study revealed that^6^left atrial size was closely correlated to left atrial/left atrial appendage thrombosis. However, it remains unclear whether the left atrial diameter combined with the CHA2DS2-VASc scoring system can improve the predictive results of left atrial/left atrial appendage thrombosis. The objective of this study was to analyze the factors related to left atrial/left atrial appendage thrombosis in patients with non-valvular AF, and explore the value of left atrial diameter combined with the CHA2DS2-VASc score in predicting left atrial/left atrial appendage thrombosis, in order to provide reference for clinical practice.

## Data and methods

### General data

This was a retrospective study. Data was collected from medical records. A total of 238 patients with non-valvular AF, who were hospitalized in Zhengzhou Cardiovascular Hospital from February 2012 to March 2017, were enrolled into the study. Inclusion criteria: (1) patients diagnosed by electrocardiogram (ECG) or dynamic ECG; (2) patients who underwent transesophageal echocardiography. Exclusion criteria: (1) patients with rheumatic heart disease, valvular AF and paroxysmal AF; (2) patients with acute myocardial infarction and acute decompensated heart failure within 90 days, and patients with previous history of cardiac surgery; (3) patients with pulmonary embolism, deep venous thrombosis, history of administration of anticoagulant drugs, such as warfarin and rivaroxaban, or lipid-lowering drugs, such as statins; (4) patients with malignant tumors, hyperthyroidism, and severe liver and kidney dysfunction. This study was approved by the Ethics Committee of our hospital. All patients provided a signed informed consent.

## Methods

### Clinical data acquisition

Gender, age, course of AF, smoking and alcohol addiction, chronic disease history, height and weight of all patients were collected, and the body mass index (BMI) was calculated. In addition, fasting blood glucose (FBG), total cholesterol (TC), triglyceride (TG), low-density lipoprotein cholesterol (LDL-C), high-density lipoprotein cholesterol (HDL-C), platelet count (Plt), serum uric acid (UA) and other biochemical indicators were collected.

### Transthoracic echocardiography and transesophageal echocardiography

All examinations were performed by an experienced sonographer with the title of Chief Physician in our hospital. All patients were routinely informed and signed an informed consent before examination. A Philips iE33 color Doppler ultrasound diagnostic apparatus was used to complete the examination. Transthoracic echocardiography was routinely performed, and the probe frequency was 2.5 MHz. Left atrial diameter (LAD), left ventricular end-diastolic dimension (LVEDD), and left ventricular ejection fraction (LVEF) were measured. Subsequently, local pharyngeal anesthesia with lidocaine was administered. Then, the probe was placed into the esophagus up to the location of the heart, and the probe frequency was 5.0 MHz. The left atrial and left atrial appendage sections were continuously observed to determine whether thrombus was present in the left atrial/left atrial appendage. These patients were divided into two groups: thrombosis group and non-thrombosis group.

### CHA2DS2-VASc score

The CHA2DS2-VASc score was calculated according to the basic clinical data of patients:^7^(1) major risk factors (2 points per item): age of ≥75 years old, ischemic stroke, and transient ischemic attack; (2) secondary factors (1 point per item): female, aged 65–74, hypertension, diabetes, vascular disease, and chronic heart failure; (3) a lowest score of 0 and a highest score of 9. The higher the score was, the greater the possibility of thrombosis.

### Statistical analysis

Data collation and statistical analyses were conducted using the statistical software SPSS 21.0. Continuous data were expressed as mean±standard deviation (x±SD), and compared between two groups using Student’s unpaired *t* -test of normal distribution, and Kolmogorov-Smirnov (K-S) test was used for normal distribution. Categorical data were expressed in rate (%), and compared between two groups using *X* ^2^-test. Multivariate logistic regression analysis was performed to analyze related factors that affected the left atrial/left atrial appendage thrombosis. The receiver operating characteristic (ROC) curve was used to analyze the predictive results of left atrial diameter and CHA2DS2-VASc score for left atrial/left atrial appendage thrombosis. p<0.05 was considered statistically significant.

## Results

### Left atrial/left atrial appendage thrombosis

A total of 238 patients with non-valvular AF were enrolled in this study. Among these patients, 151 patients were male and 87 patients were female, and the age of these patients ranged from 29 to 86, with an average age of 61.1±12.4. In these 238 patients, left atrial/left atrial appendage thrombosis occurred in 46 patients, and the incidence was 19.3%.

### Comparison of clinical data between the thrombosis group and the non-thrombosis group

Differences in gender, BMI, course of AF, proportions of patients with a history of smoking and alcohol consumption, proportions of patients with diabetes and coronary heart disease, CHA2DS2-VASc score, FBG, TC, TG, LDL-C, Plt, UA and drug therapy between the thrombosis group and non-thrombosis group were not statistically significant (p>0.05). In the thrombosis group, the proportion of patients aged ≥75, the proportion of patients with hypertension, the proportion of patients with heart failure, the proportion of patients with a history of stroke/transient ischemic attack, the proportion of patients with a history of vascular disease, and the CHA2DS2-VASc score, LAD and LVEDD were higher than those in the non-thrombosis group, while HDL-C and LVEF were lower than those in the non-thrombosis group, and all differences were statistically significant (p<0.05, Table 1 ).

**Table 1:** – Clinical Data of Thrombosis Group & Non-Thrombosis Group

Index	Thrombosis Group (n=46)	Non-Thrombosis Group (n=192)	*t* /χ^**2**^	p
Age (n, %)				
<65 yo	18 (39.1)	112 (58.3)		
65–74 yo	15 (32.6)	62 (32.3)		
≥75 yo	13 (28.3)	18 (9.4)	12.668	0.002
Sex (M/F)	32 (69.6)	119 (62.0)	0.921	0.337
BMI (kg/m^2^)	26.82±3.70	25.94±3.01	1.696	0.091
Years of AF (A)	4.69±1.69	5.10±1.38	1.718	0.087
Smoke (n, %)	18 (39.1)	71 (37.0)	0.921	0.337
Alcohol drinking (n, %)	11 (23.9)	36 (18.8)	0.624	0.429
Hypertension (n, %)	32 (69.6)	91 (47.4)	7.304	0.007
Diabetes (n, %)	8 (17.4)	44 (22.9)	0.664	0.415
Coronary disease (n, %)	4 (8.7)	8 (4.2)	1.590	0.207
HF (n, %)	7 (15.2)	6 (3.1)	10.508	0.001
Stroke/TIA (n, %)	17 (37.0)	11 (5.7)	101.138	0.000
Vascular disease (n, %)	22 (47.8)	51 (26.6)	7.890	0.005
FBG (mmol/L)	5.72±0.86	6.13±1.43	1.832	0.068
TC (mmol/L)	4.82±0.96	4.66±0.98	1.036	0.301
TG (mmol/L)	1.84±1.02	1.68±0.92	1.055	0.292
LDL-c (mmol/L)	3.00±0.54	2.96±0.86	0.298	0.766
HDL-c (mmol/L)	0.99±0.18	1.16±0.31	3.458	0.001
Plt (×10^9^/L)	209.08±34.45	214.43±41.26	0.815	0.416
UA (μmol/L)	333.70±64.68	342.74±70.08	0.798	0.426
CHA2DS2-VASc score	2.26±1.90	1.64±1.48	2.428	0.016
CHA2DS2-VASc group (n, %)			2.635	0.268
0 score	8 (17.4)	55 (28.6)		
1 score	14 (30.4)	56 (29.2)		
≥2 scores	24 (52.2)	81 (42.2)		
LAD (mm)	45.81±6.16	38.55±5.00	6.118	0.000
LVEDD (mm)	51.35±4.38	48.53±4.11	4.133	0.000
LVEF (%)	57.05±10.50	61.84±9.17	3.092	0.002
**Drug treatment (n, %)**				
β-blockers	15 (32.6)	54 (28.1)	0.362	0.547
ACEI/ARB	21 (45.7)	75 (39.1)	0.670	0.413

*BMI: body mass index; HF: heart failure; TIA: transient ischemic attack; FBG: fasting blood glucose; TC: total cholesterol; TG: triglyceride; LDL-C: low-density lipoprotein cholesterol; HDL-C: high-density lipoprotein cholesterol; Plt: platelet count; UA: serum uric acid; LAD: left atrial diameter; LVEDD: left ventricular end-diastolic dimension; LVEF: left ventricular ejection fraction; ACEI: angiotensin converting enzyme inhibitor; ARB: angiotensin receptor blockers.*

### Related factors that affect left atrial/left atrial appendage thrombosis

With the determination of whether the left atrial/left atrial appendage thrombosis existed as a dependent variable, and the variables with a p-value <0.10 as independent variables, multivariate logistic regression analysis was performed. The results revealed that the history of stroke/transient ischemic attack, vascular disease, CHA2DS2-VASc score, LAD, LVEDD and LVEF were independent risk factors for left atrial/left atrial appendage thrombosis (p<0.05, Table 2 ).

**Table 2:** – Related factors of thrombus in left atrium or left atrial appendage

Index	B	SE	Wals χ^**2**^	p	OR (95%CI)
Stroke/TIA	3.597	1.165	9.528	0.002	36.498 (3.718∼358.322)
Vascular disease	1.280	0.574	4.979	0.026	3.597 (1.168∼11.071)
HDL-c	2.574	1.021	6.354	0.012	13.124 (1.773∼97.142)
CHA2DS2-VASc score	-0.441	0.171	6.610	0.010	0.644 (0.460∼0.901)
LAD	-0.246	0.058	18.025	0.000	0.782 (0.698∼0.876)
LVEDD	-0.173	0.063	7.432	0.006	0.841 (0.743∼0.953)
LVEF	0.066	0.027	5.925	0.015	1.068 (1.013∼1.126)

*TIA: transient ischemic attack; HDL-C: high-density lipoprotein cholesterol; LAD: left atrial diameter; LVEDD: left ventricular end-diastolic dimension; LVEF: left ventricular ejection fraction.*

### The value of LAD and CHA2DS2-VASc score in predicting left atrial/left atrial appendage thrombosis

The ROC curve analysis revealed that when the CHA2DS2-VASc score was used to predict left atrial/left atrial appendage thrombosis, the area under the curve (AUC) was 0.593 (95% CI: 0.495–0.690). When the CHA2DS2-VASc score was ≥3, sensitivity and specificity were 86.5% and 32.6%, respectively. When LAD was used to predict left atrial/left atrial appendage thrombosis, the AUC was 0.786 (95% CI: 0.704–0.868). When LAD was ≥44.17 mm, sensitivity and specificity were 89.6% and 60.9%, respectively ( Figure 1 ).

**Figure 1:** – The ROC curve analysis results showed that when the CHA2DS2-VASc score was used to predict left atrial/left atrial appendage thrombosis, the area under the curve was 0.593 (95% CI: 0.495–0.690). When the CHA2DS2-VASc score was ≥3, sensitivity and specificity were 86.5% and 32.6%, respectively. When LAD was used to predict left atrial/left atrial appendage thrombosis, the area under the curve was 0.786 (95% CI: 0.704–0.868). When LAD was >44.17 mm, sensitivity and specificity were 89.6% and 60.9%, respectively.

### Effect of LAD on the risk of left atrial/left atrial appendage thrombosis in patients in the different CHA2DS2-VASc groups

In the different CHA2DS2-VASc groups, the incidence of left atrial/left atrial appendage thrombosis in patients with LAD ≥44.17 mm was higher than that in patients with LAD <44.17 mm, and the difference was statistically significant (p<0.05, Table 3 ).

**Table 3:** – The effect of left atrial diameter on the risk of thrombosis in left atrium/left atrial appendage in patients with different CHA2DS2-VASc groups (mm).

CHA2DS2-VASc group	n	left atrial/left atrial appendage thrombus (n, %)		OR	χ^**2**^	p
		LAD≥44.17	LAD<44.17			
0 score	63	6/12 (50.0)	3/51 (5.9)	8.500 (95% *CI* : 1.856∼38.938)	9.524	0.002
1 score	73	11/16 (68.8)	4/57 (7.0)	9.797 (95% *CI* : 2.747∼34.943)	15.466	0.000
≥2 score	102	11/19 (57.9)	11/83 (13.3)	4.368 (95% *CI* : 1.651∼11.559)	9.712	0.002

## Discussion

As the most common arrhythmia type in the Internal Medicine-Cardiovascular Department, AF is a risk factor that leads to thromboembolism.^8^Compared with the non-AF population, the risk of stroke in patients with AF is increased by five times.^9^In addition, a study revealed that^10^the thrombus that caused stroke in AF patients mostly came from the left atrial /left atrial appendage. Left atrial/left atrial appendage thrombosis is an independent risk factor for stroke in patients with non-valvular AF.^11^This may significantly increase the risk of thromboembolic events, and is a direct indicator of anticoagulant therapy in AF patients.^12^Therefore, early detection of left atrial/left atrial appendage thrombosis or high-risk factors for left atrial/left atrial appendage thrombosis is of great significance for guiding treatment and improving the prognosis of AF patients. In this study, 238 AF patients, who did not receive anticoagulation and lipid-lowering therapy, were enrolled. The transesophageal echocardiography results revealed that the incidence of left atrial/left atrial appendage thrombosis was 19.3%. This is similar to 18.6%, reported by Shuanglun Xie et al.,^13^and 20.7%, reported by Weiwei Fu et al.^14^These results reveal that the incidence of left atrial/left atrial appendage thrombosis is relatively high in AF patients without anticoagulation and lipid-lowering therapy.

The CHA2DS2-VASc scoring system was established by further optimizing the CHADS2 scoring system, which is a clinical method commonly used to assess the risk of stroke in AF patients at present, and also used to guide clinical treatment.^15^A study revealed that^16^a CHA2DS2-VASc score of ≥2 is an independent risk factor for left atrial/left atrial appendage thrombosis in AF patients. This study revealed that the CHA2DS2-VASc score was higher in the thrombosis group than in the non-thrombosis group. However, the difference in the distribution of CHA2DS2-VASc scores between the two groups was not statistically significant. Univariate and multivariate analysis revealed that the CHA2DS2-VASc score is an independent risk factor for left atrial/left atrial appendage thrombosis. Furthermore, these results reveal that the CHA2DS2-VASc score is correlated to left atrial/left atrial appendage thrombosis. The ROC curve analysis revealed that the AUC was 0.593 (95% CI: 0.495–0.690). When the CHA2DS2-VASc score was ≥3, sensitivity and specificity were 86.5% and 32.6%, respectively. These results showed that for patients with a CHA2DS2-VASc score of ≥3, the possibility of left atrial/left atrial appendage thrombosis should be highly alerted. However, this study also revealed that when the CHA2DS2-VASc score was 0 or 1, left atrial/left atrial appendage thrombosis still occurred in 9 and 15 patients, respectively. Furthermore, these results revealed that for low-risk patients with a CHA2DS2-VASc score of 0 or 1, there was still a risk of stroke. These results suggested that the CHA2DS2-VASc score has some limitations in predicting left atrial/left atrial appendage thrombosis.

A study revealed that^17^morphological changes in the left atrium and left atrial appendage might increase the risk of thromboembolism in AF patients. When AF occurs, the bigger the cardiac atrium, the more easily thrombosis forms.^18^In this study, LAD in AF patients was compared. The results revealed that LAD was greater in the thrombosis group than in the non-thrombosis group, which was an independent risk factor for left atrial/left atrial appendage thrombosis. The ROC curve analysis revealed that when LAD was used to predict left atrial/left atrial appendage thrombosis, the AUC was 0.786 (95% CI: 0.704–0.868), and when LAD was ≥44.17 mm, sensitivity and specificity were 89.6% and 60.9%, respectively. These results revealed that LAD size was correlated to left atrial/left atrial appendage thrombosis. Hence, when LAD was ≥44.17 mm, this had good sensitivity and specificity in predicting left atrial/left atrial appendage thrombosis. In this study, we used LAD as the index to predict left atrial/left atrial appendage thrombosis. Recently, left atrial volume has been used as a measure of left atrial enlargement.^19^This index might be included in future studies. In this study, patients were further stratified according to the CHA2DS2-VASc score, in order to analyze the effect of LAD on left atrial/left atrial appendage thrombosis. These results revealed that regardless of whether the CHA2DS2-VASc score was 0, 1 or ≥2, LAD ≥44.17 mm significantly increased the risk of left atrial/left atrial appendage thrombosis. These results revealed that further assessment of LAD on the basis of the CHA2DS2-VASc score would be helpful for evaluating the risk of left atrial/left atrial appendage thrombosis and guiding anticoagulation therapy.

However, considering that this study is a single-center, small-sample size study, there may be some shortcomings in sample representativeness. Hence, multi-center and large-sample size cohort studies are needed to further clarify the relationship between the CHA2DS2-VASc score and LAD in predicting left atrial/left atrial appendage thrombosis and guiding anticoagulation therapy.

## Conclusion

In summary, the CHA2DS2-VASc score and LAD are correlated to left atrial/left atrial appendage thrombosis in patients with non-valvular AF. For patients with a CHA2DS2-VASc score of 0 or 1, LAD size should be further considered. When LAD was ≥44.17 mm, the risk of left atrial/left atrial appendage thrombosis is still relatively high, and it is necessary to conduct further anticoagulation therapy.
